# Perceived Organizational Support, Trauma‐Informed Care, and Caring Behaviors: A Quantitative Study Among Nurses

**DOI:** 10.1155/jonm/6066488

**Published:** 2026-10-05

**Authors:** Nuriye Sarıakçalı, Sibel Polat Olca

**Affiliations:** ^1^ Department of Nursing Nursing Management Department Faculty of Health Sciences Sivas Cumhuriyet University Sivas Türkiye cumhuriyet.edu.tr; ^2^ Department of Nursing Faculty of Health Sciences Istanbul Aydin University İstanbul Türkiye aydin.edu.tr

**Keywords:** caring behaviors, nurses, nursing management, perceived organizational support, trauma-informed care

## Abstract

**Aim:**

The aim of this study is to examine the interaction between perceived organizational support, trauma‐informed care attitudes, and caring behaviors among nurses.

**Background:**

Caring behaviors are essential to nursing care quality and are influenced by both individual and organizational factors. Trauma‐informed care promotes safe, patient‐centered practices, while perceived organizational support enhances nurses’ performance and well‐being. However, their combined effect on caring behaviors remains unclear.

**Methods:**

This cross‐sectional study was conducted with 266 nurses working in a university hospital. Data were collected using a sociodemographic information form, the Caring Behaviors Inventory‐24 (CBI‐24), the Trauma‐Informed Care Scale (TICS), and the Perceived Organizational Support Scale (POSS).

**Findings:**

Nurses demonstrated high levels of caring behaviors (CBI‐24 = 5.29 ± 0.60), trauma‐informed care (TICS = 53.51 ± 7.34), and perceived organizational support (POSS = 27.95 ± 5.76). Female nurses had significantly higher caring behavior scores than males (*p* = 0.018), and nurses who received trauma‐informed care training scored higher on caring behaviors, trauma‐informed care, and perceived organizational support (*p* < 0.05). Significant positive correlations were found between caring behaviors, trauma‐informed care, and perceived organizational support (*p* < 0.05).

**Conclusions:**

Nurses’ caring behaviors are positively influenced by both trauma‐informed care and perceived organizational support. Trauma‐informed care emerged as the strongest predictor, indicating that caring behaviors are shaped not only by technical competencies but also by sensitivity to patients’ psychosocial needs and safety.

**Implications for Nursing:**

The findings underscore the important role of nursing leadership in creating organizational conditions that support both trauma‐informed care and caring practice. Nursing leaders may facilitate this process by embedding trauma‐informed principles into professional development, cultivating responsive and supportive leadership practices, promoting equitable workload allocation, and establishing effective channels of communication.

## 1. Introduction

Qualified nursing care is a fundamental determinant for patient safety and the effectiveness of healthcare services. Caring behaviors are a significant indicator reflecting nurses’ professional knowledge and skills, as well as their ethical sensitivity and patient‐centered approaches [1, 2]. Current literature emphasizes that caring behaviors are shaped not only by individual factors but also by variables such as organizational climate and managerial support [3, 4].

In addition to organizational dynamics, trauma‐informed care (TIC) stands out as a contemporary approach to improving patient experiences [5]. TIC is an approach to care that recognizes the widespread impact of trauma, acknowledges its potential effects on individuals’ health and well‐being, and integrates this understanding into care practices to promote safety, trust, collaboration, and empowerment. However, the successful implementation of TIC requires strong institutional support and managerial commitment beyond individual awareness [6, 7].

A review of the current literature reveals that perceived organizational support (POS), TIC, and caring behaviors are generally addressed as independent variables. However, studies examining the dynamic interaction between these three concepts within a holistic analytical framework are still lacking, particularly for countries with unique healthcare systems like *X*, leading to a significant gap in the existing literature.

## 2. Background

Qualified nursing care is an indispensable cornerstone in the success of healthcare services and the establishment of patient safety. The caring behaviors provided by nurses are a fundamental performance indicator that embodies not only clinical knowledge and technical skills but also empathetic abilities, ethical awareness, and patient‐centered approaches [1, 2]. Recent scientific studies emphasize that these behaviors are not limited to individual competencies alone; on the contrary, they are intertwined with the psychosocial opportunities and organizational dynamics provided by the institution [3, 4]. Current literature confirms that factors such as workload, organizational climate, emotional intelligence, and job satisfaction have a direct and decisive impact on the ways in which nurses provide care [8–10].

POS is among the leading institutional variables that are decisive for nurses’ professional well‐being and performance outcomes. Conceptually, POS represents the subjective perception developed by employees regarding the extent to which their contributions are valued by their institution and the degree to which their well‐being is cared for [11]. Current evidence in the literature confirms that a high level of POS is strongly correlated with positive outcomes such as job satisfaction, organizational commitment, and psychological well‐being, while also reinforcing the intention to stay in the organization [12–14]. Evidence from nursing populations further supports these findings, indicating that POS is significantly associated with nurses’ resilience [15].

In addition to its direct effects, organizational support also generates indirect and complementary effects through mechanisms such as self‐esteem and perceived professional benefit [16]. From a managerial perspective, POS functions as a strategic protective factor and a “buffer” mechanism that mitigates the destructive effects of chronic stressors such as workload and work–family conflict [17]. Indeed, Huang et al. [18] report that in nurses working especially in high‐risk clinical areas, a supportive organizational structure reduces the psychological deformation caused by negative experiences such as workplace violence and strengthens professional resilience.

Beyond organizational processes, the TIC approach, which has become one of the fundamental components of holistic care in contemporary healthcare, brings about a radical paradigm shift in nursing clinical practices. TIC is a strategic service model focused on preventing re‐traumatization during the care process, based on the profound impact of individuals’ past traumatic experiences on their current health status and care needs [5, 19]. Current literature emphasizes that the success of TIC practices is directly related to institutional policies and strong managerial support, in addition to the level of individual awareness [20].

Adopting a TIC‐oriented approach moves nursing care behaviors toward a more sensitive, inclusive, and holistic perspective [21]. Given the fact that nurses maintain a high frequency of contact with individuals with a history of trauma in their professional practices, there is a significant need to develop their clinical competencies and perceptions of self‐confidence in this field [22, 23]. The integration of TIC principles into clinical processes optimizes nurses’ capacity to provide empathetic, safe, and patient‐centered care [24]. However, it is reported that while internalizing TIC principles enhances patient safety and therapeutic collaboration, institutional training investments and decisive managerial support are prerequisites for the sustainability of this transformation [6, 7].

In the literature, it is emphasized that organizational support plays a crucial role in the effective implementation of TIC. Institutional training programs and managerial support significantly enhance nurses’ trauma‐sensitive care skills [7, 25]. Brennan et al. [26] reported that TIC‐based training increases health professionals’ awareness and intervention skills regarding sensitive patient groups. Studies regarding caring behaviors show that nurses are influenced by variables such as age, job satisfaction, peer relationships, and workload [27–29]. Systematic reviews reveal that organizational climate and psychological well‐being are among the important predictors of caring behaviors [4]. However, it is reported that emotional processes such as compassion fatigue and compassion avoidance negatively affect caring behaviors [10, 30]. There are also studies demonstrating the direct relationship between organizational support and quality of care. Fahimi et al. [31] showed that POS significantly increases safe care practices among intensive care nurses. Ahmad et al. [32], on the other hand, reported that organizational support has positive effects on job satisfaction and perceived quality of care.

Despite the evidence supporting these individual relationships, studies integrating POS, TIC, and caring behaviors within a unified analytical framework remain limited across healthcare settings. Examining these interrelated factors may provide a more comprehensive understanding of how organizational conditions and trauma‐informed approaches jointly shape nurses’ caring behaviors. Such evidence is particularly relevant in the context of increasing patient complexity and evolving healthcare environments and may inform nursing leaders, educators, and policymakers in developing supportive work environments, strengthening trauma‐informed education and practice, and promoting patient‐centered standards of care.

The aim of this study is to examine the interaction between POS, TIC attitudes, and caring behaviors among nurses. By testing these variables within the framework of a holistic analytical model, the study aims to provide evidence‐based insights into how institutional support and trauma‐sensitive approaches shape the quality of nursing care. Overall, the findings are expected to contribute to the international literature on nursing management and support the efforts of healthcare system stakeholders to create supportive work environments and integrate trauma‐informed paradigms into clinical practice.

The primary research question of this study is: What is the relationship between POS, TIC attitudes, and caring behaviors among nurses?

## 3. Methods

### 3.1. Research Design

The study was conducted as a cross‐sectional, quantitative study. The study period spanned from November 2025 to February 2026.

### 3.2. Sample and Setting

The data collection process was conducted between November 2025 and February 2026 at Sivas Cumhuriyet University Health Services Application and Research Hospital, a tertiary care institution in Türkiye that carries out advanced patient care, medical research, and educational activities with a holistic approach. The study population consisted of 790 nurses actively working at the institution. The sample size was calculated using the finite population sample size calculation method, based on a 95% confidence level and a 5% margin of error, indicating that a minimum of 266 participants was required [33]. During data collection, 300 nurses were invited to participate, of whom 278 completed and returned the questionnaire, yielding an initial response rate of 92.7%. After excluding 12 questionnaires with incomplete or invalid data, the final analytic sample consisted of 266 nurses, corresponding to 88.7% of those initially invited. Inclusion and exclusion criteria were clearly defined to enhance data quality and the reliability of the findings. Inclusion criteria were being a registered nurse working at the institution where the research was conducted, having at least 1 year of professional experience, and volunteering to participate in the study by providing informed consent. Exclusion criteria were defined as having less than 1 year of professional experience and refusing to participate in the study. The requirement of at least 1 year of professional experience was adopted as a methodological choice to ensure that participants had sufficient exposure to institutional operations and care processes. As a result, the final dataset included 266 participants, and no missing data were observed for any of the variables included in the analyses. A post hoc power analysis performed using G∗Power 3.1.9.7 indicated that, with a sample size of 266 and an alpha level of 0.05, the achieved statistical power (1 − *β*) ranged from 0.96 to 0.99 for the observed effect sizes (*f* ^2^ = 0.053–0.089). These findings confirm that the sample size was adequate for the analyses conducted.

### 3.3. Variables and Instruments

#### 3.3.1. Descriptive Characteristics Information Form

This data collection form, developed by the researchers, was designed to determine the sociodemographic and professional characteristics of the participants. It consists of questions addressing variables such as gender, age, education level, marital status, unit of employment (emergency department, intensive care unit, internal medicine clinics, surgical clinics, outpatient clinics, administrative units, operating room, and delivery room), and duration of professional experience.

#### 3.3.2. Perceived Organizational Support Scale (POSS)

The original scale was developed by Eisenberger et al. [34], and later reduced to 10 items by Armstrong‐Stassen and Ursel [35]. The Turkish validity and reliability of the scale were established by Akkoç et al. [36]. Validity and reliability studies specific to a nursing sample in Türkiye were conducted by Türe and Yıldırım [37]. The scale consists of nine items and a single subdimension and is structured as a 5‐point Likert‐type scale. An increase in the total score obtained from the scale indicates a higher level of POS, and the scale does not have a defined cut‐off point. In the study by Türe and Yıldırım [37], the Cronbach’s alpha coefficient was reported as 0.88.

#### 3.3.3. Trauma‐Informed Care Scale (TICS)

The scale was developed by King et al. [38], and consists of 21 items and three subdimensions. The Turkish validity and reliability study was conducted by Tekin and Bayar [21]; based on the results of their analysis, three items were removed from the attitude subdimension, resulting in a final version consisting of 18 items and three subdimensions. There are no reverse‐scored items in the Turkish version. The scale evaluates the TIC levels (knowledge, attitudes, and practices) of mental health professionals working with individuals who have a history of trauma. It is scored on a 5‐point Likert‐type scale ranging from 0 to 4. Cronbach’s alpha coefficients were reported as 0.81 for practice, 0.72 for knowledge, 0.82 for attitude, and 0.80 for the total scale. There is no cutoff score; a low score indicates a need for further knowledge, while a high score reflects a high level of TIC [21].

#### 3.3.4. Caring Behaviors Inventory‐24 (CBI‐24)

CBI‐24 was developed by Wu et al. [39] as a short form of the 42‐item Caring Behaviors Inventory‐42 (CBI‐42) developed by Wolf et al. [40], and it is utilized to evaluate the nursing care process. In addition to allowing for the comparison of nurses’ self‐assessments with patient perceptions, the scale is also used to evaluate care behaviors provided during the pre and postoperative periods (such as listening, education, and involving the patient in the decision making process). The CBI‐24 consists of a total of 24 items across four subdimensions: assurance (8 items), knowledge and skills (5 items), respectfulness (6 items), and connectedness (5 items), and it is scored on a 6‐point Likert type scale (1 = Never, 6 = Always). In the original study of the scale, the Cronbach’s alpha coefficient was found to be 0.96 for the total scale and between 0.82 and 0.92 for the subdimensions. The Turkish validity and reliability study was conducted by Kurşun and Kanan [41], with Cronbach’s alpha values determined as 0.97 for patients and 0.96 for nurses. The correlations of the subdimensions with the total scale ranged between 0.85 and 0.94 in the patient group and between 0.81 and 0.95 in the nurse group. The total scale score is calculated by dividing the sum of all items by 24, while subdimension scores are calculated by taking the average of the relevant items. Scores range from 1 to 6, with higher scores indicating that caring behaviors are perceived at a more positive level [41].

### 3.4. Data Collection

Before commencing the data collection process, necessary administrative permissions were obtained from the Sivas Cumhuriyet University Health Services Application and Research Hospital where the research was conducted. Following the institutional approval, meetings were held with the head nurses working in different clinics and units of the hospital to ensure an effective and organized data collection process, and an implementation plan was established. The researchers contacted the nurses who met the inclusion criteria individually, provided a brief briefing regarding the aim and scope of the study, and subsequently obtained their written informed consent. Following the acquisition of informed consents, the data collection tools were prepared in both printed and electronic formats. Printed questionnaires were delivered to the participants by hand to be completed face to face at their convenience; for nurses who preferred online participation, the survey link was transmitted electronically. This approach was adopted to increase the accessibility of participants and to facilitate the data collection process. Participants completed the questionnaires during their break times to avoid disrupting clinical operations, and the data collection period was planned to take approximately 20–30 min.

### 3.5. Ethical Considerations

The ethical appropriateness of the study was ensured through approval obtained from the Sivas Cumhuriyet University Health Sciences Research Ethics Committee (Date: October 30, 2025; Decision No: 2025‐10/02). In addition, the necessary institutional permissions were secured from the university hospital administration prior to data collection. The study was conducted in accordance with the principles of the Declaration of Helsinki. Participants were informed in detail about the purpose and scope of the study, as well as the principle of voluntary participation. Participation was entirely voluntary, and written informed consent was obtained from all nurses included in the study. To ensure confidentiality, personal identifying information was not collected; instead, unique numerical codes were assigned to each participant, and all data were analyzed anonymously. Furthermore, participants were informed that they could withdraw from the study at any time without providing any reason and without facing any consequences.

### 3.6. Data Analyses

The research data were analyzed using the IBM SPSS Statistics 25.0 software package. Continuous variables were presented as mean ($\overset{¯}{x}$), standard deviation (SD), minimum (min.), and maximum (max.) values, while categorical variables were expressed as frequency (*n*) and percentage (%). The normality of continuous variables was assessed by evaluating multiple criteria simultaneously. In this context, skewness and kurtosis coefficients were considered within ±1.0 as ideal and within ±2.0 as acceptable limits [42, 43]. In addition, *Q*–*Q* plot graphs were visually examined, and it was determined that the distribution patterns were consistent with these criteria [44]. Based on these findings, the data were assumed to meet the normality assumption, and parametric tests were preferred in the analyses. In group comparisons, independent samples *t*‐test, Mann–Whitney *U* test, ANOVA, and Kruskal–Wallis H test were used, considering the distribution characteristics of the variables. For group comparisons, effect sizes were calculated using Cohen’s *d* for parametric tests, rank‐biserial correlation (*r*) for the Mann–Whitney *U* test, eta squared (*η* ^2^) for one‐way ANOVA, and epsilon squared (*ε* ^2^) for the Kruskal–Wallis test. Partial *R* ^2^ values were reported for each predictor in the hierarchical regression model. The construct validity of the three scales was examined using confirmatory factor analysis (CFA). Given the ordinal nature of the Likert‐type items, CFA was performed using the weighted least squares mean and variance adjusted (WLSMV) estimator. Model fit was evaluated using the comparative fit index (CFI), Tucker–Lewis index (TLI), root mean square error of approximation (RMSEA), and standardized root mean square residual (SRMR). A three‐step hierarchical multiple linear regression analysis was conducted to determine the factors affecting nurses’ CBI‐24 total score. In this model, sociodemographic variables were included in the first block, the POSS in the second block, and the total score of the TICS in the third block. Assumptions related to the regression analysis were evaluated as follows: multicollinearity (VIF < 5.0), autocorrelation (Durbin–Watson coefficient between 1.5 and 2.5), influence of outliers (Cook’s distance < 1.0), and normality of residuals (assessed via histogram and scatter plots) [44, 45]. The statistical power of the study was calculated using the G∗Power 3.1.9.7 program [46]. The mediating role of TIC was examined using Hayes’ PROCESS macro (Model 4) with 5000 bootstrap resamples, controlling for gender and years of professional experience. The reliability of the scales was evaluated using Cronbach’s alpha coefficient, and the level of statistical significance was set at *p* < 0.05 for all analyses.

## 4. Results

### 4.1. Participant Characteristics

Table 1 presents the distribution of nurses’ sociodemographic characteristics. The mean age of the nurses was 36.94 years, and the mean duration of professional experience was 14.44 years. It was determined that 82.7% of the nurses were female, 64.7% held a bachelor’s degree, and 66.2% were married. When the units of employment were examined, 31.6% were working in internal medicine clinics. In terms of receiving training on TIC, 63.5% of the nurses had not received any training (Table 1).

**TABLE 1:** Distribution of descriptive characteristics of the nurses (*n* = 266).

Characteristics	$\overset{¯}{x}$ ± SD (*n*)	Min.–Max. (%)
Age	36.94 ± 9.26	21–60
Years of work experience	14.44 ± 10.17	1–39
Gender		
Female	220	82.7
Male	46	17.3
Education		
High school	41	15.4
Bachelor’s degree	172	64.7
Master’s degree and above	53	19.9
Marital status		
Married	176	66.2
Single	90	33.8
Unit of employment		
Emergency department	16	6.0
Operating room	16	6.0
Surgical clinic	34	12.8
Internal medicine clinic	84	31.6
Outpatient clinic	35	13.2
Intensive care unit	43	16.2
Management (charge nurse/administrative unit)	38	14.3
Training on trauma‐informed care		
Yes	97	36.5
No	169	63.5

*Note:* $\overset{¯}{x}$, mean; *n*, number; %, percentage; Min, minimum value; Max, maximum value.

Abbreviation: SD, standard deviation.

### 4.2. Mean Scores and Reliability Coefficients of the Caring Behaviors, TICS, and POSS

Table 2 presents the descriptive statistics for the scales and their subdimensions. Examination of the mean scores of the CBI‐24 revealed that the assurance subdimension had a mean of 5.28 (SD = 0.64), knowledge–skill subdimension 5.35 (SD = 0.63), respectful subdimension 5.23 (SD = 0.62), connectedness subdimension 5.29 (SD = 0.63), and the total score 5.29 (SD = 0.60). For the TICS, the mean scores were 19.30 (SD = 2.84) for the knowledge subdimension, 15.92 (SD = 3.34) for the attitude subdimension, 18.29 (SD = 3.57) for the practice subdimension, and 53.51 (SD = 7.34) for the total score. The mean total score of the POSS was found to be 27.95 (SD = 5.76). Cronbach’s alpha reliability coefficients are also presented in the table (Table 2).

**TABLE 2:** Descriptive statistics for the CBI‐24, TICS, and POSS scales and their subscales.

Scales	$\overset{¯}{x}$ ± SS	Min.–Max.	Cronbach alpha (*α*)
CBI‐24	5.29 ± 0.60	3.29–6	0.968
Assurance	5.28 ± 0.64	3.13–6	0.943
Knowledge and skill	5.35 ± 0.63	3.20–6	0.900
Respectful	5.23 ± 0.62	3.17–6	0.835
Connectedness	5.29 ± 0.63	3.40–6	0.845
TICS	53.51 ± 7.34	34–72	0.850
Knowledge	19.30 ± 2.84	6–24	0.801
Attitude	15.92 ± 3.34	7–24	0.685
Practice	18.29 ± 3.57	2–24	0.906
POSS	27.95 ± 5.76	13–45	0.745

*Note:* $\overset{¯}{x}$, mean; Min, minimum value; Max, maximum value.

Abbreviations: CBI‐24, Caring Behaviors Inventory‐24; POSS, Perceived Organizational Support Scale; SD, standard deviation; TICS, Trauma‐Informed Care Scale.

#### 4.2.1. Construct Validity of the Scales

CFA showed CFI = 0.967, TLI = 0.963, RMSEA = 0.113, and SRMR = 0.064 for the CBI‐24; CFI = 0.952, TLI = 0.944, RMSEA = 0.126, and SRMR = 0.096 for the TICS; and CFI = 0.934, TLI = 0.908, RMSEA = 0.192, and SRMR = 0.067 for the POSS. The fit indices showed a mixed pattern, with generally acceptable CFI and TLI values but elevated RMSEA values; therefore, the CFA findings were interpreted cautiously.

### 4.3. Comparison of Caring Behaviors, TIC, and POS According to Nurses’ Characteristics

As shown in Table 3, caring behaviors differed significantly by gender, with female nurses reporting higher scores across all CBI‐24 subdimensions and the total score than male nurses (*p* < 0.05), although the corresponding effect sizes were small. Marital status was associated only with the TICS attitude subdimension, with single nurses reporting higher scores than married nurses (*p* = 0.008; *d* = 0.348). No significant differences were observed according to educational level (*p* > 0.05). TIC training was associated with higher scores across several outcomes. Nurses who had received training reported significantly higher CBI‐24 knowledge and skill, respectful, and connectedness subdimension scores, as well as higher total CBI‐24 scores (*p* < 0.05), with small effect sizes. They also had higher TICS attitude, practice, and total scores and higher POSS scores (*p* < 0.05). The largest difference was observed for the TICS attitude subdimension (*d* = 0.667), representing a moderate effect. No significant difference was observed in the TICS knowledge subdimension (*p* = 0.856). Detailed group comparisons, effect sizes, and 95% confidence intervals are presented in Table 3.

**TABLE 3:** Comparison of the mean subscale and total scores of the CBI‐24, TICS, and POSS according to nurses’ characteristics.

Characteristics	*n*	Assurance	Knowledge and skill	Respectful	Connectedness	CBI‐24	Knowledge	Attitude	Practice	TICS	POSS
		$\overset{¯}{x}$ ± SD	$\overset{¯}{x}$ ± SD	$\overset{¯}{x}$ ± SD	$\overset{¯}{x}$ ± SD	$\overset{¯}{x}$ ± SD	$\overset{¯}{x}$ ± SD	$\overset{¯}{x}$ ± SD	$\overset{¯}{x}$ ± SD	$\overset{¯}{x}$ ± SD	$\overset{¯}{x}$ ± SD
Gender											
Female	220	5.32 ± 0.62	5.40 ± 0.61	5.28 ± 0.61	5.33 ± 0.61	5.33 ± 0.58	19.20 ± 2.78	15.97 ± 3.46	18.47 ± 3.48	53.64 ± 7.55	27.70 ± 6.00
Male	46	5.10 ± 0.67	5.13 ± 0.66	5.00 ± 0.64	5.10 ± 0.69	5.08 ± 0.63	19.83 ± 3.14	15.67 ± 2.72	17.41 ± 3.88	52.91 ± 6.29	29.17 ± 4.34
Test		*Z* = −2.105	*Z* = −2.674	*Z* = −2.884	*Z* = −2.124	*Z* = −2.370	*Z* = −0.982	*t* = 0.542	*Z* = −1.673	*t* = 0.607	*Z* = −1.867
*p*		0.035	0.008	0.004	0.034	0.018	0.326	0.588	0.094	0.544	0.062
ES		*r* = 0.195	*r* = 0.247	*r* = 0.269	*r* = 0.197	*r* = 0.222	*r* = 0.091	*d* = 0.088	*r* = 0.155	*d* = 0.098	*r* = 0.175
%95 CI (ES)		(0.014; 0.364)	(0.068; 0.410)	(0.092; 0.430)	(0.016; 0.366)	(0.042; 0.388)	(−0.092; 0.268)	(−0.230; 0.406)	(−0.028; 0.327)	(−0.220; 0.416)	(−0.007; 0.345)
Education											
High school	41	5.37 ± 0.53	5.46 ± 0.56	5.26 ± 0.63	5.32 ± 0.56	5.35 ± 0.52	19.07 ± 3.05	16.00 ± 3.76	18.32 ± 3.82	53.39 ± 7.37	28.22 ± 7.51
Bachelor’s degree	172	5.24 ± 0.65	5.33 ± 0.62	5.22 ± 0.62	5.28 ± 0.64	5.26 ± 0.60	19.24 ± 2.97	15.84 ± 3.36	17.96 ± 3.59	53.04 ± 7.54	27.97 ± 5.40
Master’s degree and above	53	5.33 ± 0.66	5.34 ± 0.69	5.25 ± 0.64	5.31 ± 0.65	5.31 ± 0.63	19.68 ± 2.20	16.11 ± 3.00	19.34 ± 3.09	55.13 ± 6.54	27.70 ± 5.48
Test		*H* = 1.187	*H* = 1.061	*H* = 0.319	*H* = 0.185	*H* = 0.710	*H* = 1.929	*F* = 0.152	*H* = 5.883	*F* = 1.658	*H* = 0.110
*p*		0.553	0.588	0.852	0.912	0.701	0.381	0.859	0.053	0.193	0.947
ES		*ε* ^2^ = 0.004	*ε* ^2^ = 0.004	*ε* ^2^ = 0.001	*ε* ^2^ = 0.001	*ε* ^2^ = 0.003	*ε* ^2^ = 0.007	*η* ^2^ = 0.001	*ε* ^2^ = 0.022	*η* ^2^ = 0.012	*ε* ^2^ = 0.000
Marital status											
Married	176	5.29 ± 0.62	5.35 ± 0.61	5.24 ± 0.63	5.30 ± 0.62	5.29 ± 0.58	19.38 ± 3.01	15.53 ± 3.25	18.08 ± 3.61	52.99 ± 7.43	27.83 ± 5.62
Single	90	5.26 ± 0.68	5.36 ± 0.66	5.23 ± 0.62	5.28 ± 0.63	5.28 ± 0.62	19.16 ± 2.51	16.68 ± 3.41	18.70 ± 3.45	54.53 ± 7.10	28.20 ± 6.06
Test		*t* = 0.314	*t* = −0.109	*t* = 0.169	*t* = 0.273	*t* = 0.191	*t* = 0.647	*t* = −2.683	*Z* = −1.417	*t* = −1.628	*t* = −0.495
*p*		0.754	0.913	0.866	0.785	0.848	0.519	0.008	0.157	0.105	0.621
ES		*d* = 0.041	*d* = 0.014	*d* = 0.022	*d* = 0.035	*d* = 0.025	*d* = 0.079	*d* = 0.348	*r* = 0.105	*d* = 0.211	*d* = 0.064
%95 CI (ES)		(−0.213; 0.295)	(−0.240; 0.268)	(−0.232; 0.276)	(−0.219; 0.289)	(−0.229; 0.279)	(−0.175; 0.333)	(0.092; 0.603)	(−0.042; 0.247)	(−0.044; 0.465)	(−0.190; 0.318)
Training on trauma‐informed care											
Yes	97	5.38 ± 0.57	5.46 ± 0.55	5.38 ± 0.51	5.39 ± 0.55	5.40 ± 0.52	19.21 ± 2.76	17.27 ± 2.92	19.07 ± 3.11	55.55 ± 6.95	28.95 ± 5.30
No	169	5.22 ± 0.67	5.29 ± 0.66	5.15 ± 0.67	5.23 ± 0.66	5.22 ± 0.63	19.36 ± 2.90	15.14 ± 3.33	17.84 ± 3.74	52.34 ± 7.33	27.38 ± 5.96
Test		*t* = 1.937	*t* = 2.096	*t* = 3.048	*t* = 2.058	*t* = 2.342	*Z* = −0.181	*t* = 5.423	*t* = 2.746	*t* = 3.496	*t* = 2.144
*p*		0.054	0.037	0.003	0.041	0.020	0.856	< 0.001	0.006	< 0.001	0.033
ES		*d* = 0.247	*d* = 0.267	*d* = 0.362	*d* = 0.262	*d* = 0.298	*r* = 0.013	*d* = 0.667	*d* = 0.350	*d* = 0.445	*d* = 0.273
%95 CI (ES)		(−0.004; 0.497)	(0.016; 0.517)	(0.110; 0.613)	(0.011; 0.513)	(0.047; 0.549)	(−0.130; 0.156)	(0.410; 0.922)	(0.098; 0.601)	(0.192; 0.697)	(0.022; 0.524)

*Note:* $\overset{¯}{x}$, mean; *t*, independent samples *t*‐test value; *Z*, standardized *Z* value of the Mann–Whitney *U* test; *F*, one‐way analysis of variance (ANOVA) test value; H, Kruskal–Wallis *H* test value. For comparisons presented in two rows, the first row shows the effect size and the second row shows the 95% CI. *d*, Cohen’s *d* effect size; *r*: rank‐biserial correlation coefficient; *η* ^2^, eta squared; *ε* ^2^, epsilon squared. Bonferroni correction was applied to the between‐group comparisons.

Abbreviations: CBI‐24, Caring Behaviors Inventory‐24; CI: confidence interval; POSS, Perceived Organizational Support Scale; SD, standard deviation; TICS, Trauma‐Informed Care Scale.

### 4.4. Correlation Between Nurses’ Age and Years of Experience and Their Caring Behaviors, TIC, and POS Levels

Table 4 presents the relationships between nurses’ age and years of experience and the subdimension and total scores of the CBI‐24 and TICS, as well as the total score of the POSS.

**TABLE 4:** Relationship between nurses’ age and years of experience and the subscale and total scores of the CBI‐24 and TICS, and the total score of the POSS.

No	Variables	Test; *p*	1	2	3	4	5	6	7	8	9	10	11	12
1	Age	*r*	1											
		*p*												

2	Years of work experience	*r*	0.953^∗∗^	1										
		*p*	< 0.001											

3	Assurance	*r*	0.116	0.138^∗^	1									
		*p*	0.059	0.024										

4	Knowledge and skill	*r*	0.079	0.092	0.889^∗∗^	1								
		*p*	0.197	0.134	< 0.001									

5	Respectful	*r*	0.145^∗^	0.137^∗^	0.837^∗∗^	0.850^∗∗^	1							
		*p*	0.018	0.025	< 0.001	< 0.001								

6	Connectedness	*r*	0.101	0.113	0.835^∗∗^	0.894^∗∗^	0.890^∗∗^	1						
		*p*	0.100	0.066	< 0.001	< 0.001	< 0.001							

7	CBI‐24	*r*	0.119	0.130^∗^	0.952^∗∗^	0.953^∗∗^	0.940^∗∗^	0.945^∗∗^	1					
		*p*	0.053	0.034	< 0.001	< 0.001	< 0.001	< 0.001						

8	Knowledge	*r*	0.130^∗^	0.135^∗^	0.072	0.025	0.012	0.041	0.043	1				
		*p*	0.035	0.027	0.242	0.688	0.850	0.507	0.485					

9	Attitude	*r*	−0.020	−0.057	0.241^∗∗^	0.161^∗∗^	0.206^∗∗^	0.185^∗∗^	0.215^∗∗^	0.218^∗∗^	1			
		*p*	0.743	0.351	< 0.001	0.009	0.001	0.003	< 0.001	< 0.001				

10	Practice	*r*	0.128^∗^	0.110	0.239^∗∗^	0.188^∗∗^	0.229^∗∗^	0.192^∗∗^	0.228^∗∗^	0.223^∗∗^	0.556^∗∗^	1		
		*p*	0.037	0.074	< 0.001	0.002	< 0.001	0.002	< 0.001	< 0.001	< 0.001			

11	TICS	*r*	0.103	0.080	0.254^∗∗^	0.174^∗∗^	0.209^∗∗^	0.193^∗∗^	0.225^∗∗^	0.595^∗∗^	0.810^∗∗^	0.825^∗∗^	1	
		*p*	0.093	0.196	< 0.001	0.004	0.001	0.002	< 0.001	< 0.001	< 0.001	< 0.001		

12	POSS	*r*	0.065	0.038	0.158^∗∗^	0.119	0.187^∗∗^	0.084	0.149^∗^	−0.038	0.245^∗∗^	0.206^∗∗^	0.197^∗∗^	1
		*p*	0.293	0.533	0.010	0.052	0.002	0.172	0.015	0.539	< 0.001	0.001	0.001	

*Note:* Pearson correlation coefficient (*r*).

Abbreviations: CBI‐24, Caring Behaviors Inventory‐24; TICS, Trauma‐Informed Care Scale; POSS, Perceived Organizational Support Scale.

^∗^ *p* < 0.05.

^∗∗^ *p* < 0.01.

^∗∗∗^ *p* < 0.001.

Age and years of experience showed weak positive correlations with selected dimensions of caring behaviors and TIC (*r* = 0.128–0.145, *p* < 0.05). Caring behavior dimensions were positively associated with the attitude and practice dimensions of TIC, with correlation coefficients ranging from 0.161 to 0.241 (*p* < 0.01). POS was positively associated with the total CBI‐24 score (*r* = 0.149, *p* = 0.015) and with TICS attitude (*r* = 0.245, *p* < 0.00 1), practice (*r* = 0.206, *p* = 0.001), and total scores (*r* = 0.197, *p* = 0.001). Overall, the observed associations were positive but generally weak. Detailed correlation coefficients are presented in Table 4.

### 4.5. The Effect of POS and TIC on Nurses’ Caring Behaviors: A Hierarchical Regression Analysis

Table 5 presents the hierarchical regression analysis examining the predictors of nurses’ caring behaviors. Age was excluded because of its high correlation with years of experience (*r* = 0.953, *p* < 0.001), while years of experience and gender were entered in the first block. POSS and TICS total scores were subsequently added in the second and third blocks, respectively. The first model explained 2.9% of the variance in CBI‐24 scores (adj. *R* ^2^ = 0.029, *p* = 0.008), with female gender emerging as a significant predictor. Adding POS significantly increased the explained variance (Δ*R* ^2^ = 0.026, *p* = 0.008), and the inclusion of TIC produced a further significant increase (Δ*R* ^2^ = 0.034, *p* = 0.002). In the final model, TIC was the strongest predictor of caring behaviors (*β* = 0.189, *p* = 0.002), followed by female gender (*β* = 0.150, *p* = 0.013) and POS (*β* = 0.124, *p* = 0.042). The final model explained 8.2% of the variance (adj. *R* ^2^ = 0.082), while years of experience was not a significant predictor. Regression assumptions were satisfied, with no evidence of residual autocorrelation, influential outliers, or multicollinearity (Durbin–Watson = 1.945; maximum Cook’s *D* = 0.059; maximum VIF = 1.054). Detailed regression coefficients and model statistics are presented in Table 5. As a sensitivity analysis, the TICS subscales were entered simultaneously as separate predictors. The model yielded an adjusted *R* ^2^ of 0.082, and none of the subscales independently predicted caring behaviors (knowledge: *p* = 0.895; attitude: *p* = 0.073; practice: *p* = 0.137). No problematic multicollinearity was observed (maximum VIF = 1.557) (Supporting Table S1).

**TABLE 5:** The effect of perceived organizational support and trauma‐informed care level on nurses’ caring behaviors: Hierarchical regression analysis.

Models	Independent variables	Unstandardized coefficients		Standardized coefficients	*t*	*p*	Partial *R* ^2^	95.0% CI for *B*		Collinearity statistics	
		*B*	Std. Error	Beta (*β*)				LB	UB	Tolerance	VIF
1	Constant	5.013	0.095	—	52.931	< 0.001		4.826	5.199		
	Years of work experience	0.006	0.004	0.106	1.723	0.086	0.011	−0.001	0.013	0.971	1.030
	Gender = female	0.222	0.097	0.141	2.301	0.022	0.020	0.032	0.413	0.971	1.030

2	Constant	4.532	0.202	—	22.389	< 0.001		4.134	4.931		
	Years of work experience	0.006	0.004	0.097	1.590	0.113	0.010	−0.001	0.013	0.968	1.033
	Gender (ref.: male)										
	Female	0.249	0.096	0.159	2.596	0.010	0.025	0.060	0.438	0.960	1.041
	POSS	0.017	0.006	0.161	2.676	0.008	0.027	0.004	0.029	0.988	1.013

3	Constant	3.843	0.297	—	12.945	< 0.001		3.258	4.428		
	Years of work experience	0.005	0.004	0.085	1.412	0.159	0.008	−0.002	0.012	0.964	1.038
	Gender (ref.: male)										
	Female	0.236	0.095	0.150	2.493	0.013	0.023	0.050	0.422	0.958	1.044
	POSS	0.013	0.006	0.124	2.045	0.042	0.016	< 0.001	0.025	0.949	1.054
	TICS	0.015	0.005	0.189	3.131	0.002	0.036	0.006	0.025	0.954	1.048

	**Model fit and summary**										

Model 1	*F* (2, 263) = 4.951. *p* = 0.008; *R* = 0.190; *R* ^2^ = 0.036; Adj. *R* ^2^ = 0.029.										
Model 2	*F* (3, 262) = 5.766. *p* = 0.001; *R* = 0.249; *R* ^2^ = 0.062; Adj. *R* ^2^ = 0.051; Δ*R* ^2^ = 0.026; Δ*F* (1, 262) = 7.162, *p* = 0.008.										
Model 3	*F* (4, 261) = 6.920. *p* < 0.001; *R* = 0.249; *R* ^2^ = 0.096; Adj. *R* ^2^ = 0.082; Δ*R* ^2^ = 0.034; Δ*F* (1, 261) = 9.801, *p* = 0.002; DW = 1.945; Cook’s *D* = 0.059 < 1.0; VIF_max_ = 1.054 < 5.0										

	**Dependent variable: CBI-24**										

*Note: B*, unstandardized regression coefficient; Std., standard; LB–UB, lower and upper bounds of the 95% confidence interval for *B*; beta (*β*), standardized regression coefficient; *R* ^2^, coefficient of determination (explained variance); Adj. *R* ^2^, adjusted *R* ^2^; DW, Durbin–Watson autocorrelation coefficient.

Abbreviations: CBI‐24, Caring Behaviors Inventory‐24; POSS, Perceived Organizational Support; TICS, Trauma‐Informed Care Scale; VIF, variance inflation factor.

### 4.6. Mediating Role of TIC in the Relationship Between POS and Caring Behaviors

Table 6 presents the results of the mediation analysis examining the mediating role of TIC in the relationship between POS and caring behaviors. The mediation analysis was conducted with years of work experience and gender (female; reference category: male) included as covariates. POS had a positive and statistically significant effect on TIC (path *a*: *B* = 0.253, *β* = 0.199, *p* = 0.001, 95% CI [0.101, 0.406]). After controlling for POS and the covariates, TIC also had a positive and statistically significant effect on caring behaviors (path *b*: *B* = 0.015, *β* = 0.189, *p* = 0.002, 95% CI [0.006, 0.025]). The total effect of POS on caring behaviors was statistically significant (path *c*: *B* = 0.017, *β* = 0.161, *p* = 0.008, 95% CI [0.004, 0.029]). After TIC was included in the model, the direct effect decreased but remained statistically significant (path *c′*: *B* = 0.013, *β* = 0.124, *p* = 0.042, 95% CI [0.000, 0.025]). The indirect effect of POS on caring behaviors through TIC was also positive, and the 95% confidence interval based on 5000 bootstrap samples did not include zero (*B* = 0.004, BootSE = 0.002, 95% bootstrap CI [0.001, 0.008]; *β* = 0.038). These findings are consistent with a partial mediating role of TIC in the relationship between POS and caring behaviors after accounting for years of work experience and gender as covariates (Table 6). The mediation model illustrating the mediating role of TIC in the relationship between POS and caring behaviors is presented in Figure 1 (Figure 1).

**TABLE 6:** The mediating role of trauma‐informed care in the relationship between perceived organizational support and caring behaviors.

Predictor variables	Outcome variables									
	*M* (mediator variable)					*Y* (dependent variable)				
	TICS					CBI‐24				
	Path	Coeff.	SE	*β*	*p*	Path	Coeff.	SE	*β*	*p*
POSS	*a*	0.253	0.077	0.199	0.001	*c*′	0.013	0.006	0.124	0.042
TICS						*b*	0.015	0.005	0.189	0.002
Constant	iM	45.025	2.515		< 0.001	iY	3.843	0.297		< 0.001

	*R* ^2^ = 0.046					*R* ^2^ = 0.096				
	*F* (3,262) = 4.212, *p* = 0.006					*F* (4,261) = 6.920, *p* < 0.001				

	**Effect**	**SE**		**LLCI**		**ULCI**	**β**		**p**	

Total effect (*c* path)	0.017	0.006		0.004		0.029	0.161		0.008	
Direct effect (*c*′ path)	0.013	0.006		0.000		0.025	0.124		0.042	

	**Effect**	**BootSE**		**BootLLCI**		**BootULCI**	**β**			

Indirect effect (*a* × *b* path)	0.004	0.002		0.001		0.008	0.038			

*Note:* Coeff., unstandardized regression coefficient; *β*, standardized regression coefficient; *R* ^2^, coefficient of determination (explained variance); *F*, ANOVA F statistic; LLCI, lower limit of the 95% confidence interval; ULCI, upper limit of the 95% confidence interval; BootLLCI, lower limit of the 95% bootstrap confidence interval; BootULCI, upper limit of the 95% bootstrap confidence interval. The indirect effect was estimated using 5000 bootstrap samples; *n* = 266. Years of work experience and gender (female; reference category: male) were included in the mediation analysis as covariates.

Abbreviations: BootSE, bootstrap standard error; CBI‐24, Caring Behaviors Inventory‐24; POSS, Perceived Organizational Support; SE, Standard error; TICS, Trauma‐Informed Care Scale.

**FIGURE 1:**
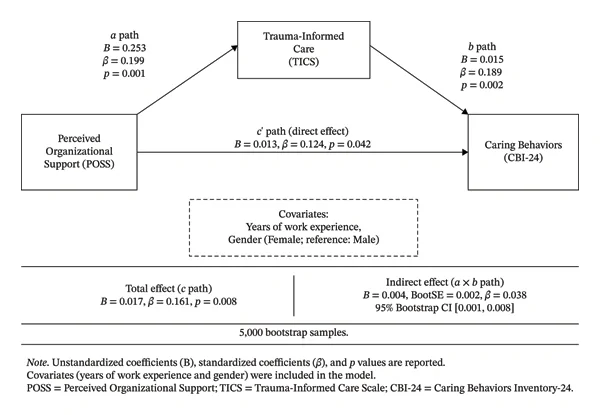
Mediating role of trauma‐informed care in the relationship between perceived organizational support and caring behaviors. *Note:* Unstandardized coefficients (*B*), standardized coefficients (*β*), and *p* values are reported. Covariates (years of work experience and gender) were included in the model. POSS, Perceived Organizational Support; TICS, Trauma‐Informed Care Scale; CBI‐24, Caring Behaviors Inventory‐24.

## 5. Discussion

This study examines the factors affecting nurses’ caring behaviors within the context of TIC levels and POS. The findings revealed that the mean scores of the CBI‐24 and its subdimensions were generally high. This result is consistent with previous studies reporting that nurses’ caring behaviors are predominantly at moderate‐to‐high levels [4, 27–29]. Similarly, studies conducted in different clinical settings indicate that caring behaviors are influenced by professional experience, individual characteristics, the work environment, and organizational factors [2, 3, 8].

In this context, the high level of caring behaviors observed in the present study suggests that nurses have adopted a patient‐centered care approach and strongly reflect the dimensions of assurance, respect, knowledge–skill, and connectedness in the care process. The study also found that female nurses had higher mean scores in the CBS subdimensions and total score compared to male nurses. This finding is consistent with some studies indicating that caring behaviors may be associated with gender. In particular, it has been reported that the dimensions of care related to empathy, sensitivity, and emotional support may be more prominent among female nurses [2, 8]. However, the literature indicates that findings regarding gender are not consistent, and some studies report no significant difference in caring behaviors between female and male nurses [1]. Therefore, when interpreting the gender‐related findings of the present study, it should be taken into account that the majority of the sample consisted of female participants.

One of the important findings of this study is that nurses who received TIC training had higher levels of caring behaviors, TIC attitude and practice scores, as well as POS scores. TIC is a holistic approach that considers individuals’ trauma histories and prioritizes safety, empowerment, collaboration, and sensitivity [5, 19]. Previous studies have demonstrated that TIC training improves nurses’ knowledge, attitudes, and practices, and contributes to delivering care in a more sensitive, safe, and patient‐centered manner [7, 20, 22, 25, 26, 47]. In line with these findings, the results of the present study suggest that TIC training can be an important tool both for strengthening caring behaviors and for improving the quality of clinical care.

Significant positive correlations were found between age and years of experience and some subdimensions of caring behaviors and TIC. The weak strength of these relationships suggests that experience alone does not fully explain caring behaviors; however, as clinical experience increases, skills such as communication, decision‐making, recognizing patient needs, and managing the care process may improve. The literature also indicates that experience, professional maturity, and clinical awareness may positively influence caring behaviors [3, 8, 48]. However, it is also reported that caring behaviors are not determined solely by experience but are associated with variables such as workload, burnout, compassion fatigue, emotional intelligence, and psychological well‐being [10, 30, 49].

In this study, positive relationships were found between POS and both caring behaviors and TIC levels. POS refers to employees’ perceptions that they are valued by their organization and that their well‐being is cared for. Studies in the field of nursing indicate that organizational support has positive effects on job satisfaction, work engagement, psychological well‐being, resilience, safe care, and quality of care [11, 12, 15, 16, 31, 50]. The current findings also demonstrate that a supportive organizational climate strengthens nurses’ caring behaviors and may positively influence TIC practices.

The results of the hierarchical regression analysis revealed that the level of TIC was the strongest predictor of the total CBI‐24 score. This finding indicates that caring behaviors are not solely based on technical knowledge and skills, but are also shaped by a sensitive care approach that considers patients’ psychosocial needs, trauma history, and need for safety. Strengthening TIC in nursing practice may contribute to the delivery of ethical, safe, and patient‐centered care, particularly for vulnerable patient groups [5, 6, 19, 23, 51].

POS was also found to significantly predict caring behaviors in the regression model. This finding is consistent with studies indicating that organizational support enhances nurses’ professional performance, work engagement, and quality of care [17, 52–55]. In addition, POS has been reported to play a protective role against burnout, job stress, and turnover intention [13, 14, 56–58]. From a nursing management perspective, these findings are particularly relevant because nurse managers influence many of the organizational conditions through which support is experienced in everyday practice, including communication, recognition, participation in decision‐making, workload management, and psychological safety. Supportive and responsive leadership may therefore create conditions that enable nurses to engage more consistently in patient‐centered and TIC practices. Accordingly, strengthening managerial practices that promote equitable workload allocation, open communication, staff participation, recognition, and psychological safety may represent an important organizational strategy for supporting nurses’ caring behaviors.

However, the relatively low proportion of variance explained by the model indicates that caring behaviors have a multidimensional structure. Caring behaviors may be influenced by numerous factors such as individual characteristics, clinical experience, work environment, workload, organizational climate, professional satisfaction, compassion fatigue, secondary traumatic stress, and burnout [2, 10, 30, 49]. The clinical context of the participants should also be considered when interpreting these findings. Nurses working across different clinical units may encounter substantially different working conditions in terms of patient acuity, workload, staffing demands, frequency of exposure to trauma‐related situations, and emotional demands of care. Therefore, the distribution of participants across clinical settings may have influenced their perceptions of organizational support, TIC, and caring behaviors. Although clinical unit was not examined as a contextual or clustering factor in the present analyses, these potential unit‐level differences should be considered when interpreting the findings. Future multicenter studies incorporating unit‐level characteristics and organizational, psychological, and clinical factors into more comprehensive analytical models may provide a more nuanced understanding of the determinants of nurses’ caring behaviors and how clinical environments shape these relationships.

A key finding of this study was the partial mediating role of TIC in the relationship between POS and caring behaviors. This finding suggests that organizational support may be associated with caring behaviors not only directly but also through nurses’ trauma‐informed attitudes and practices. Supportive and psychologically safe work environments may facilitate the integration of trauma‐informed principles into clinical practice, thereby promoting more sensitive and patient‐centered caring behaviors. The persistence of a significant direct association suggests that other organizational and psychological mechanisms may also contribute to this relationship.

In conclusion, the findings of this study indicate that as nurses’ levels of TIC and POS increase, their caring behaviors also improve. The widespread implementation of TIC training may strengthen nurses’ knowledge, attitudes, and practices in this area. At the same time, creating supportive work environments may enhance both nurses’ professional well‐being and the quality of care. However, since some findings were at the threshold of statistical significance and the explained variance remained limited, the results should be interpreted with caution and supported by further studies with larger samples, multicenter designs, and longitudinal approaches.

### 5.1. Limitations

This study has several limitations that should be considered when interpreting the findings. First, the cross‐sectional design limits the ability to draw causal inferences regarding the relationships between POS, TIC, and nurses’ caring behaviors. The findings reflect associations at a single point in time and do not indicate the directionality of these relationships.

Second, this study was conducted at a single healthcare institution in Türkiye, and the sample was predominantly female. These characteristics may limit the generalizability of the findings to more diverse populations, healthcare settings, and countries, particularly where organizational, cultural, and clinical conditions differ. Future multicenter and cross‐national studies involving more diverse samples are needed to assess the broader applicability of these findings.

Third, the use of self‐report measures may have introduced response and social desirability bias, particularly for caring behaviors and TIC. In addition, although data were collected using both paper‐based and online questionnaires, the mode of administration was not recorded at the individual level, precluding assessment of potential mode effects. Furthermore, demographic and professional data were unavailable for nonrespondents; therefore, comparisons with respondents could not be performed, and potential selection bias cannot be entirely excluded.

Fourth, the lack of assessment of factors that may influence caring behaviors, such as shift patterns, workload, and burnout, is a limitation of this study.

Future research with larger and more diverse samples, as well as multicenter and longitudinal designs, is recommended to provide stronger evidence.

### 5.2. Implications for Nursing Management and Future Research

The findings of this study provide important implications for nursing management and future research. Considering that TIC and POS significantly influence nurses’ caring behaviors, nursing managers should prioritize strategies aimed at strengthening these areas. In particular, the systematic implementation of TIC training programs may enhance nurses’ knowledge, attitudes, and practices, thereby improving the quality of care.

Enhancing POS is also highly important from a managerial perspective. Supportive leadership, open communication, fair workload distribution, and encouraging staff participation in decision‐making processes may increase nurses’ organizational commitment and motivation. Such practices may contribute not only to employee well‐being but also to improved patient care quality and safety.

The finding that female nurses demonstrated higher levels of caring behaviors suggests that individual differences should be considered in care delivery. In this context, nursing management should develop inclusive training and support programs aimed at strengthening the caring competencies of all nurses.

For future research, it is recommended that factors influencing caring behaviors be examined more comprehensively. Including variables such as workload, burnout, leadership style, organizational climate, and psychological well‐being may contribute to the development of more robust explanatory models. In addition, the use of longitudinal and experimental designs is recommended to better clarify causal relationships among variables. Multicenter studies conducted across different institutional and cultural contexts would enhance the generalizability of findings. Furthermore, qualitative and mixed‐methods studies may provide deeper insights into nurses’ experiences.

Overall, further research in this area is essential for improving both managerial practices and the scientific understanding of caring behaviors.

## 6. Conclusions

This study demonstrates that nurses’ caring behaviors are significantly associated with both TIC levels and POS. The findings indicate that higher levels of TIC and organizational support are linked to more positive caring behaviors, highlighting the importance of both individual competencies and organizational context in shaping nursing care.

Among the variables examined, TIC emerged as the strongest predictor of caring behaviors, suggesting that care practices are not solely based on technical knowledge and skills but are also influenced by a sensitive, patient‐centered approach that considers patients’ psychosocial needs, trauma histories, and safety. In addition, POS was found to be a significant contributor, emphasizing the role of supportive work environments in enhancing nurses’ professional performance and quality of care.

These results underline the need to strengthen TIC competencies through structured training programs and to foster supportive organizational climates characterized by effective leadership, open communication, and fair working conditions. Such improvements may contribute to both enhanced nurse well‐being and higher‐quality, safer patient care.

However, given the cross‐sectional design and the limited variance explained by the model, the findings should be interpreted with caution. Future studies using larger, more diverse samples and longitudinal or experimental designs are recommended to further clarify causal relationships and expand the understanding of factors influencing caring behaviors.

Overall, this study contributes to the growing body of evidence emphasizing the importance of integrating trauma‐informed approaches and organizational support strategies into nursing practice to improve care outcomes.

## Funding

No funding was received for this manuscript.

## Conflicts of Interest

The authors declare no conflicts of interest.

## Supporting Information

Additional supporting information can be found online in the Supporting Information section.

## Supporting information


**Supporting Information 1** Supporting Table S1: Sensitivity analysis: regression model with TICS subscales predicting nurses’ caring behaviors (*n* = 266).

**Supporting Information 2** STROBE Statement—Checklist of items that should be included in reports of cross‐sectional studies.

## Data Availability

The data that support the findings of this study are available from the corresponding author upon reasonable request.
