# Supplementary material for: Perceived Organizational Support, Trauma‐Informed Care, and Caring Behaviors: A Quantitative Study Among Nurses
Source: J Nurs Manag. 2026 Oct 5;2026:6066488. doi: 10.1155/jonm/6066488 (PMC13639548; doi:10.1155/jonm/6066488)
Supplement: Supplementary file 2 [file JONM-2026-6066488-s002.docx]

**Supplementary Table**

**Supplementary Table S1** Sensitivity analysis: regression model with TICS subscales predicting nurses’ caring behaviors (n=266)

| **Independent variables** | **B** | **Std. Error** | **Beta (β)** | **t** | **p** | **95.0% CI for B (LB)** | **95.0% CI for B (UB)** | **Tolerance** | **VIF** |
| --- | --- | --- | --- | --- | --- | --- | --- | --- | --- |
| Constant | 4.052 | 0.332 | - | 12.214 | <0.001 | 3.399 | 4.706 |  |  |
| Years of work experience | 0.006 | 0.004 | 0.100 | 1.621 | 0.106 | -0.001 | 0.013 | 0.916 | 1.092 |
| Gender=Female (ref.: Male) | 0.212 | 0.096 | 0.135 | 2.208 | 0.028 | 0.023 | 0.402 | 0.925 | 1.081 |
| POSS | 0.011 | 0.006 | 0.104 | 1.670 | 0.096 | -0.002 | 0.023 | 0.900 | 1.111 |
| TICS Knowledge | -0.002 | 0.013 | -0.008 | -0.133 | 0.895 | -0.027 | 0.024 | 0.888 | 1.126 |
| TICS Attitude | 0.024 | 0.013 | 0.132 | 1.799 | 0.073 | -0.002 | 0.049 | 0.642 | 1.557 |
| TICS Practice | 0.018 | 0.012 | 0.109 | 1.491 | 0.137 | -0.006 | 0.042 | 0.649 | 1.540 |
| **Model Fit and Summary** | | | | | | | | | |
| F(6, 259)=4.950. p<0.001; R=0.321; R^2^=0.103; Adj. R^2^=0.082; DW=1.855; Cook's D=0.091<1.0; VIFmax=1.557<5.0 | | | | | | | | | |
| Dependent variable: CBI-24 | | | | | | | | | |

B: Unstandardized regression coefficient; Std.: Standard; LB–UB: Lower and upper bounds of the 95% confidence interval for B; Beta (β): Standardized regression coefficient; R²: Coefficient of determination (explained variance); Adj. R²: Adjusted R²; VIF: Variance inflation factor; DW: Durbin–Watson autocorrelation coefficient.

Abbreviation: CBI-24, Caring Behaviors Inventory-24; TICS, Trauma-Informed Care Scale; POSS, Perceived Organizational Support Scale.
